# Immunoregulation and genotype in colorectal cancer

**DOI:** 10.1186/2051-1426-3-S2-P282

**Published:** 2015-11-04

**Authors:** Sarah E Church, Gabriela Bindea, Bernhard Mlecnik, Tessa Fredricksen, Jerome Galon

**Affiliations:** 1 INSERM, Paris, France

## 

Classical methods for determining malignant disease prognosis are based upon characterizing the morphology and location of tumor cells. While this analysis provides important information about a patient's disease it fails to capture the biological complexity of the tumor microenvironment and the contribution of the anti-tumor immune response. Basic histological quantification of T lymphocyte density, cytotoxicity, and memory by CD3, CD8, and CD45RO, respectively, has demonstrated that increased infiltration of T lymphocytes is associated with statistically significant improvement in patients' disease-free survival (DFS) and overall survival (OS) in colorectal carcinoma (CRC). Delineating the location of T lymphocytes into two areas within the primary tumor, the center and the invading margin is termed Immunoscore.

In this study we examined whether tumor genotype could predict the clinical outcome of patients with CRC and if combining Immunoscore with analysis of the tumor genotype could further enhance the accuracy of predicting clinical outcome. We analyzed 200 primary colorectal tumor samples for mutations in 50 common driver genes, including those implicated in models of colon carcinogenesis (APC, KRAS, BRAF, SMAD4). Each mutation was then assessed for prediction of DFS. Patients presented with a mean of 4 ± 2 mutations. The most common mutations were in KRAS (58%), TP53 (49%) and APC (37%). Neither, a single mutation or the number of mutations significantly predicted clinical outcome.

We examined whether mutations in the primary tumor were associated with presence of metastasis. Mutational profiles were remarkably similar between primary tumors from patients whether or not they had metastatic progression at the time of surgery. None of the 50 major cancer genes was more frequently mutated in patients with metastasis. However, using integrative analysis we found that tumors from patients with metastatic disease downregulated adaptive immune genes including those involved in co-stimulation, proliferation and positive regulation of T cells. Furthermore when analyzing the ability to predict patients' OS using Immunoscore in these late stage tumors, high Immunoscore still distinguished patients with better clinical prognosis. These data suggest immune contexture accurately predicts clinical outcome regardless of tumor stage. Further analysis of pre-existing immunity together with the analysis of tumor genotype may provide better understanding of tumor progression and invasion.

